# Differences in the Pathogenicity and Inflammatory Responses Induced by Avian Influenza A/H7N9 Virus Infection in BALB/c and C57BL/6 Mouse Models

**DOI:** 10.1371/journal.pone.0092987

**Published:** 2014-03-27

**Authors:** Guangyu Zhao, Chenfeng Liu, Zhihua Kou, Tongtong Gao, Ting Pan, Xiaohong Wu, Hong Yu, Yan Guo, Yang Zeng, Lanying Du, Shibo Jiang, Shihui Sun, Yusen Zhou

**Affiliations:** 1 State Key Laboratory of Pathogen and Biosecurity, Beijing Institute of Microbiology and Epidemiology, Beijing, China; 2 Lindsley F. Kimball Research Institute, New York Blood Center, New York, United States of America; 3 Key Laboratory of Medical Molecular Virology of Ministries of Education and Health, Shanghai Medical College and Institute of Medical Microbiology, Fudan University, Shanghai, China; Chinese Academy of Medical Sciences, China

## Abstract

Avian influenza A/H7N9 virus infection causes pneumonia in humans with a high case fatality rate. However, virus-induced modulation of immune responses is being recognized increasingly as a factor in the pathogenesis of this disease. In this study, we compared the pathogenicity of A/H7N9 infection in BALB/c and C57BL/6 mouse models, and investigated the putative involvement of proinflammatory cytokines in lung injury and viral clearance. In both mouse strains, A/Anhui/1/2013(H7N9) infection with 10^6^ TCID_50_ resulted in viral replication in lung, severe body weight loss and acute lung injury. During the early infection stage, infected C57BL/6 mice exhibited more severe lung injury, slower recovery from lung damage, less effective viral clearance, higher levels of interlukine (IL)-6, monocyte chemotactic protein (MCP)-1, and IL-1β, and lower levels of tumor necrosis factor (TNF)-α and interferon (IFN)-γ than infected BALB/c mice. These results suggest that TNF-α and IFN-γ may help suppress viral gene expression and increase viral clearance, and that IL-6 and MCP-1 may contribute to lung injury in A/H7N9-infected individuals. In addition, lung damage and the distribution of virus antigen in tissues were similar in young and middle-aged mice. These results suggest that the more serious lung injury in middle-aged or older H7N9 cases is not mainly caused by differences in viral replication in the lung but probably by a dysregulated immune response induced by underlying comorbidities. These results indicate that the extent of dysregulation of the host immune response after H7N9 virus infection most probably determines the outcome of H7N9 virus infection.

## Introduction

During March 2013, a novel avian influenza A/H7N9 virus was identified in Shanghai and Anhui, China [1]. By November 6, 2013, 139 laboratory confirmed human cases of A/H7N9 infection, including 45 deaths (a case fatality rate of 32%), had been reported to the World Health Organization [2]. Most of the A/H7N9-infected patients suffered from pneumonia, but some of them exhibited acute respiratory distress syndrome (ARDS) with respiratory failure [1].

The dysregulation of proinflammatory cytokines and chemokines, or a “cytokine storm”, a severe adverse reaction created by the secretion of large amounts of proinflammatory cytokines, may aggravate lung injury observed in A/H5N1- and A/H7N9-infected patients [3], [4]. Chi et al. [5] reported that the serum concentrations of IFNγ-inducible protein 10 (IP-10), IL-6, IL-17, and IL-2 were significantly higher in A/H7N9-infected patients than in normal individuals and that those of IP-10 and IL-6 were much higher in severe A/H7N9 patients than in non-sever patients. Chen et al. [6] showed that the serum IL-10 level in a patient who died from A/H7N9 infection was much higher than that in a patient who survived A/H7N9 infection. Zhou et al. [7] also found that the levels of IP-10, monokine induced by γ interferon (MIG), macrophage inflammatory protein 1 beta (MIP-1β), MCP-1, IL-6, IL-8 and IFN-α were significantly higher in patients with A/H7N9 than in healthy subject controls. With the exception of MIG and MIP-1β levels, there were no significant differences in the levels of these cytokines and chemokines in patients infected with A/H7N9 or H5N1 viruses. Mok et al. [8] reported that A/H7N9-infected BALB/c mice exhibited mild, self-limited disease with higher lung titers of H7N9 virus and higher serum levels of several proinflammatory cytokines and chemokines during the early stage of viral infection. However, the cytokines potentially involved in lung injury and viral clearance are not known.

Clinical data show that the fatality risks of patients admitted to hospital differ substantially depending on age. Increasing age is associated with greater disease severity in patients infected with seasonal influenza [9]. In contrast to the skewed age distribution of young people with highly pathogenic avian (HPAI) influenza A H5N1 virus infection, middle-aged or older patients with underlying medical conditions were among the severe cases of H7N9 virus infection. However, it is still unclear whether disease severity of patients is related to their susceptibility to H7N9 virus infection or to differences in the activities of host factors after virus infection.

BALB/c and C57BL/6 mice have been used widely to study the pathogenesis of infectious diseases and exhibit different susceptibilities and immune responses to invading pathogens. For example, Otte et al. [10] reported that C57BL/6 mice were more susceptible to pH1N1 influenza virus infection than BALB/c mice and that HPAI H5N1 virus was more virulent in BALB/c mice than in C57BL/6 mice. C57BL/6 mice show increased Th1 activity and a strong Th1 cytokine response upon infection. BALB/c mice show increased Th2-type cell activity [11]. In the present study, we used BALB/c and C57BL/6 mice infected with A/Anhui/1/2013(H7N9) influenza virus to study the pathogenesis of A/H7N9 infection in two strains and to determine the potential effects of proinflammatory cytokine dysregulation.

## Materials and Methods

### Ethics statement

All of the procedures involving animals were approved by the Laboratory Animal Center, State Key Laboratory of Pathogen and Biosecurity, Beijing Institute of Microbiology and Epidemiology (Permit number BIME 2013-15). The animal studies were conducted in strict accordance with the recommendations in the Guide for the Care and Use of Laboratory Animals. All of the procedures involving live A/H7N9 viruses were carried out in an approved biosafety level 3 facility.

### Mice and viruses

Specific pathogen-free young C57BL/6 and BALB/c mice (6-week-old males and females) and female middle-aged BALB/c mice (10 months old) were obtained from the Laboratory Animal Center, Academy of Military Medical Sciences, Beijing, China. A/Anhui/1/2013 (H7N9) virus (Anhui/1/H7N9) was initially isolated by the National Institute for Viral Diseases Control and Prevention, Chinese Center for Diseases Control and Prevention (CDC) from a throat-swab specimen of the third case of laboratory-confirmed A/H7N9 virus [1], and was authorizedly provided by Chinese CDC. Anhui/1/H7N9 virus used in this study was harvested-allantoic fluid following two passages in Madin-Darby canine kidney (MDCK) cells and two subsequent propagations in the allantoic cavity of embryonated eggs. The sequence of the virus was confirmed by sequencing the entire genome using a Genome Sequencer Junior (Roche).

### Experimental infection of C57BL/6 mice and BALB/c mice

The juvenile C57BL/6 and BALB/c mice and the middle-aged BALB/c mice were anesthetized via intraperitoneal injection of ketamine (80 mg/kg) and then inoculated intranasally (i.n.) with a 50% tissue culture infective dose (TCID_50_) of 10^6^ H7N9 virus in a volume of 20 μl. The infected mice were sacrificed at the times indicated and the lung, spleen, liver, kidney, brain, and intestine were harvested for virological and pathological analyses. Mouse sera were also collected for cytokine analysis. In addition, the young C57BL/6 and BALB/c mice were monitored daily to assess weight loss following H7N9 virus infection.

### Analysis of serum cytokines

Sera were collected from the infected mice at the times indicated and stored at −20°C before being analyzed. The levels of IL-1β, TNF-α, IL-6, IFN-γ, MCP-1, and keratinocyte-derived cytokine (KC) (a mouse homologue of IL-8) in the mouse sera were determined quantitatively using an enzyme-linked immunosorbent assay (ELISA) (Neobioscience, China), according to the manufacturer's protocol. Briefly, 100 μl of diluted mouse serum was added to a plate precoated with a monoclonal antibody specific for individual mouse cytokines and incubated at 37°C for 90 min. After washing, biotinylated-detection antibodies specific to each cytokine were added and incubated at 37°C for 1 h. Plates were then washed and incubated for 1 h with 100 μl of HRP-conjugate streptavidin. The reactions were developed with 3,3′,5,5′-tetramethylbenzidine (TMB) substrate (Invitrogen, Carlsbad, CA) at 37°C for 30 min, followed by adding 1 N H_2_SO_4_ to stop the reactions. Plates were then read on an ELISA plate reader (Synergy 2, BioTek) at 450 nm. The levels of mouse serum cytokines were determined using standard curves.

### Viral titers in lung tissues

The tissues of infected mice were harvested at the times indicated and homogenized in minimal essential medium (MEM) plus antibiotics to produce 10% (w/v) suspensions. The viral titers in tissues were determined as the TCID_50_, as described previously [12]. In brief, monolayers of MDCK cells were inoculated with tenfold serial dilutions of mouse organ homogenates in quadruplicate. Two hours after inoculation, the supernatants were removed and replaced with MEM plus antibiotics and 2 μg/ml TPCK-trypsin (Sigma, Shanghai, China). The viral cytopathic effect was observed for 3 days before viral infectivity in MDCK cells was measured using a hemagglutination assay with 0.5% turkey erythrocytes. The tissue viral titers were calculated using the Reed and Muench method and expressed as log_10_ TCID_50_/g of tissue [13].

### Histological analysis of lung damage

A portion of lung tissue was excised, fixed with 10% neutral buffered formalin, dehydrated, and then embedded in paraffin before performing routine histology. Sections measuring 6 μm in thickness were stained with hematoxylin and eosin (H&E) and examined by light microscopy. The extent of lung injury was scored according to the levels of degeneration and necrosis of the bronchi and bronchiolar epithelium, infiltration of inflammatory cells, alveoli degeneration and collapse, expansion of parenchymal wall, hemorrhage, and interstitial edema [14].

### Immunohistochemical staining of a neutrophil marker and the hemagglutinin (HA) protein of H7N9 virus in lung tissue

Formalin-fixed, paraffin-embedded lung sections were de-paraffinized with xylene and hydrated using a graded alcohol series. The changes in the levels of HA protein of A/H7N9 virus and infiltrating neutrophils were assessed using a rabbit polyclonal antibody to A/H7N9 HA (Sino Biological Inc., China) and neutrophil marker NIMP-R14 (Santa Cruz Biotechnology Inc, Santa Cruz, CA), respectively. Antibodies were detected using a standard streptavidin-biotin detection system (Beijing Zhongshan Biotechnology Co. Ltd, Beijing, China), according to the manufacturer's instructions.

Neutrophil infiltration was assessed semi-quantitatively by examining in a blinded manner for the presence of neutrophils in 10 arbitrarily selected 40× objective fields of lung parenchyma in each lung section by light microscopy. The cumulative scores for each animal were expressed as the number of positive fields per 100 fields (%) [14].

### Statistical analysis

All of the analyses were performed using Graphpad Prism version 5.01. The differences in the gross lesion areas between mouse strains were analyzed using a nonparametric Mann-Whitney test. The differences between mouse groups at the times indicated were analyzed using a two-way ANOVA and a Bonferroni post test.

## Results

### Lung injury in A/H7N9-infected C57BL/6 and BALB/c mice

**Figure 1:**
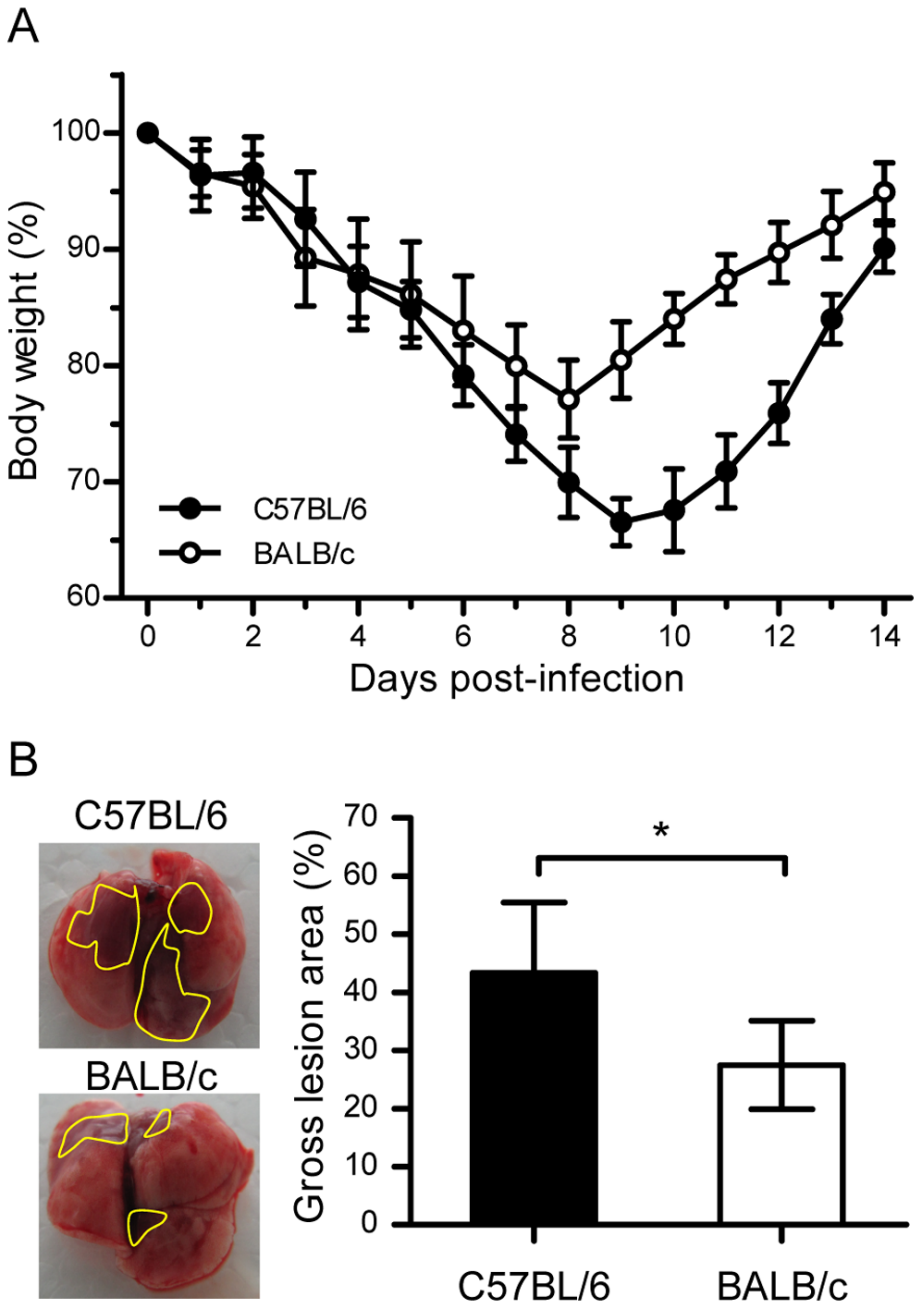
Weight change and gross pathology of the lungs in C57BL/6 and BALB/c mice infected with H7N9 virus. (A) Weight changes in C57BL/6 and BALB/c mice after challenge with AH/1/H7N9 virus. Healthy young C57BL/6 and BALB/c mice (equal numbers of males and females) were infected intranasally with 10^6^ TCID_50_ of AH1/H7N9 virus and monitored for 14 days. The body weights were measured daily (*n* = 12) and the data are expressed as the mean ± standard deviation (SD). (B) Gross pathology of the lungs from C57BL/6 and BALB/c mice at day 3 postviral infection. The gross lesion area percentage is indicated on the right (*n* = 6) and the data are expressed as the mean ± SD (bar). *, **, and *** indicate *P*<0.05, 0.01, and 0.001, respectively.

To determine whether the pathogenicity H7N9 virus infection differed between mouse strains, healthy 6-week-old C57BL/6 and BALB/c mice were infected i.n. with 10^6^ TCID_50_ of AH/1/H7N9 virus. Both C57BL/6 and BALB/c mice exhibited ruffled fur and hunched backs during the days following infection (data not shown). Although none of the mice died after infection with AH/1/H7N9 virus, body weight loss was clearly observed in all of the infected mice. Compared with BALB/c mice, which had an average body weight loss of 25% on day 8 postviral infection, C57BL/6 mice exhibited more severe weight loss, particularly on days 9–10 postviral infection. Moreover, infected C57BL/6 mice recovered body weight loss more slowly than infected BALB/c mice (Fig. 1A).

During necropsy, the gross pathologies of the lungs from the two mouse strains after A/H7N9 virus infection were similar with respect to multifocal consolidation and dark red discolorations, but the gross lesion areas were significantly different (*P*<0.05), with mean gross lesion areas of 28% and 45% in BALB/c mice and C57BL/6 mice, respectively. Gross presentations in both mice after A/H7N9 virus infection indicated more severe lung damage in C57BL/6 mice than in BALB/c mice (Fig. 1B).

**Figure 2:**
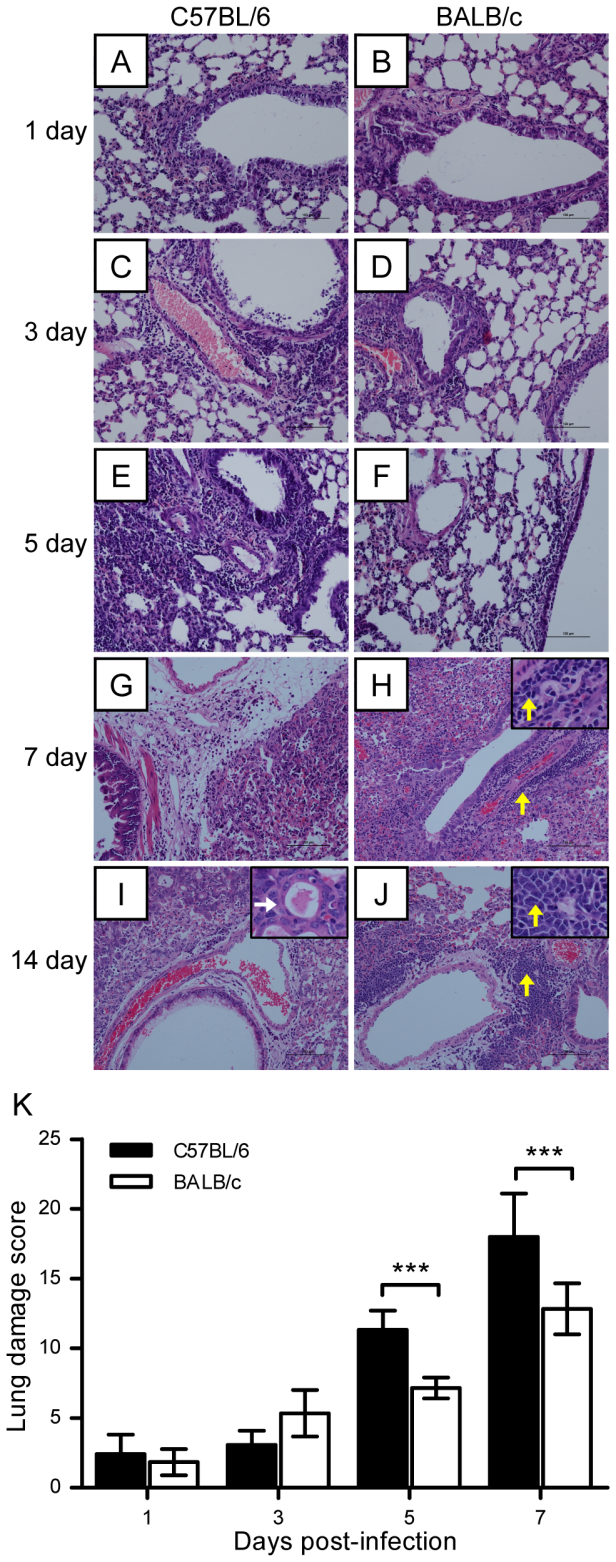
Lung injury in C57BL/6 and BALB/c mice following H7N9 virus infection. C57BL/6 and BALB/c mice were challenged intranasally with 10^6^ TCID_50_ of AH1/H7N9 virus and euthanized on days 0, 1, 3, 5, 7, and 14 after viral infection. (A–J) Histological changes in mouse lung tissues on days 0, 1, 3, 5, 7, and 14 postviral challenge. White and yellow arrows indicate the regeneration of pneumocytes and dissociation cells, respectively. (K) Semiquantitative assessments of lung lesions in mice on days 1, 3, 5, and 7 postviral infection (*n* = 6). The data are expressed as the mean ± SD (bar). *, **, and *** indicate *P*<0.05, 0.01, and 0.001, respectively.

Histopathological analysis of mouse lung, spleen, brain, intestine, and kidney at different time points after AH/1/H7N9 virus infection showed no significant changes in the histology of mouse spleen, brain, intestine, or kidney. However, significant lung injury was detected in C57BL/6 and BALB/c mice infected with AH/1/H7N9. On days 1 and 3 postviral infection, lung damage in the form of vacuolated bronchial epithelial cells on day 1 and necrosis on day 3 was similar in C57BL/6 and BALB/c mice. In addition, a patchy inflammatory response was observed around the bronchioles and blood vessels (Fig. 2A–D). On days 5 and 7 postviral infection, lung damage was noticeably more severe in C57BL/6 mice than in BALB/c mice, as evidenced by alveolar and interstitial flooding of inflammatory cells, severe widening of lung septa, interstitial edema, fibrin, focal hemorrhage in parenchyma, and scattered bronchiolar and terminal airway epithelial degeneration and necrosis (Fig. 2E–H), as well as a higher lung damage score (Fig. 2K). In particular, on day 7 postviral infection, regeneration of the bronchial epithelium and cells around blood vessels was observed in the lungs of BALB/c mice, but not in the lungs of C57BL/6 mice (Fig. 2H). On day 14 postviral infection, the parenchyma regeneration was observed in both strains, but cell proliferation was higher around the blood vessels of BALB/c mice (Fig. 2I–J). These results demonstrate that C57BL/6 mice were more susceptible to A/H7N9 infection than BALB/c mice, and that A/H7N9-infected C57BL/6 mice had more severe lung injury than A/H7N9-infected BALB/c mice. Furthermore, BALB/c mice exhibited more rapid restoration of lung tissue than C57BL/6 mice.

**Figure 3:**
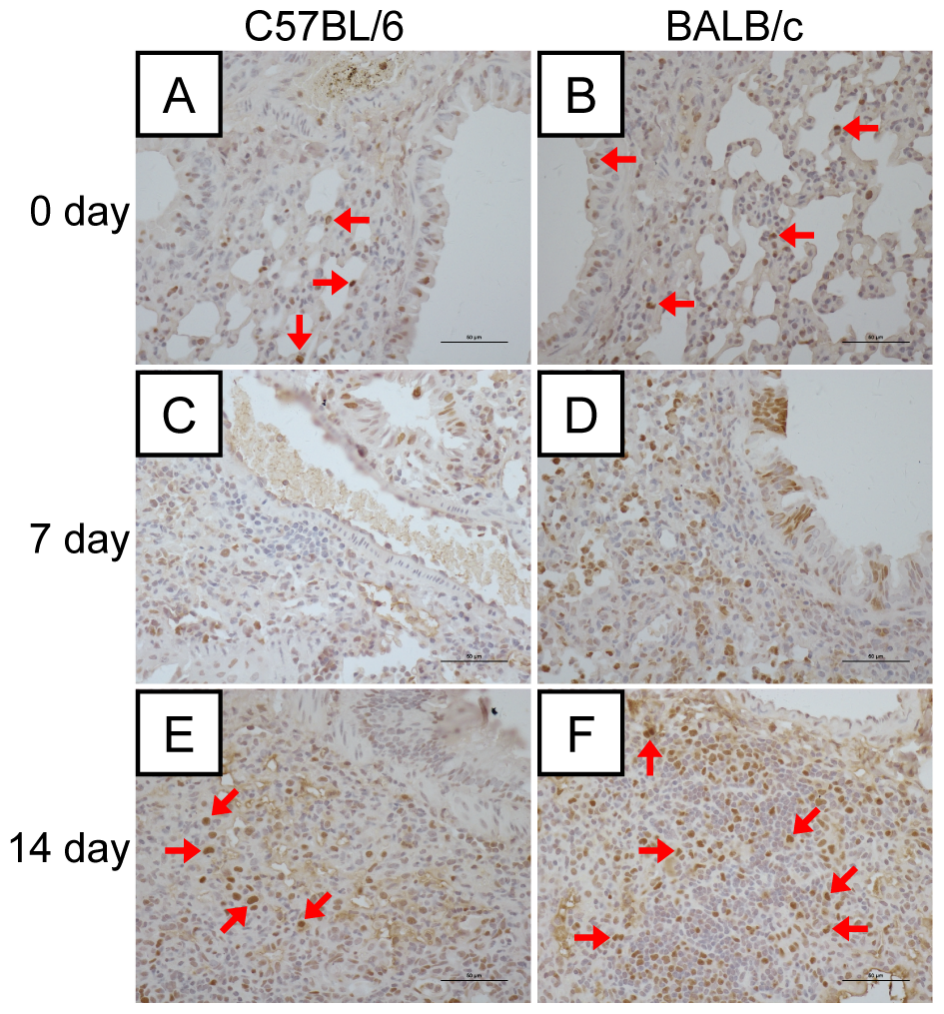
Regeneration of lung tissues in mice following H7N9 virus infection. (A–F) Immunohistochemical staining of proliferating cell nuclear antigen (PCNA)-positive cells in the lung tissues of C57BL/6 and BALB/c mice on days 0, 7, and 14 postviral infection. Positive cells were more abundant in the bronchial epithelial cells and interstitial tissues in the lungs. Red arrows indicate PCNA-positive cells.

To further investigate and compare the recovery of C57BL/6 and BALB/c mice from A/H7N9 virus infection, we stained cells for proliferating cell nuclear antigen (PCNA), an indicator of cell proliferation. The number of PCNA-positive cells was similar in the two strains of mice at 0, 3, and 5 days after virus infection (data not shown). On days 7 and 14 after viral infection, however, the number of regenerative cells among bronchial epithelial cells and in the interstitial tissues of the lungs of BALB/c mice was higher than that in the lungs of C57BL/6 mice (Fig. 3), thereby further confirming that BALB/c mice recover more effectively than C57BL/6 mice from A/H7N9 infection.

### Virus infection and viral clearance in C57BL/6 mice and BALB/c mice

**Figure 4:**
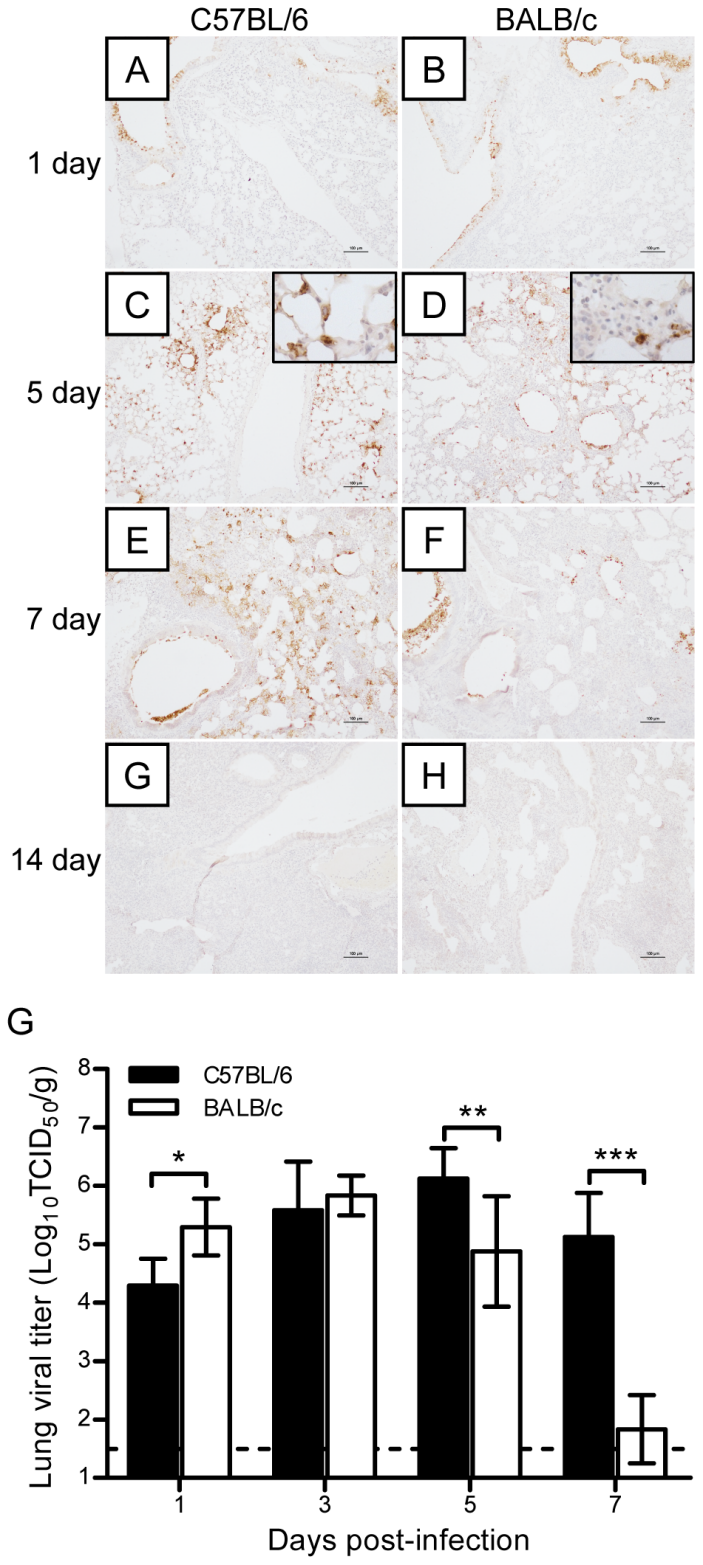
H7N9 virus antigen distribution in lung tissues after H7N9 virus infection. (A–H) Immunohistochemical staining of H7N9 virus antigen in the lungs of C57BL/6 and BALB/c mice on days 1, 5, 7, and 14 postviral infection. (I) Dynamic changes in the viral titers in the lungs of C57BL/6 and BALB/c mice on days 1, 3, 5, and 7 postviral infection (*n* = 6). The data are expressed as the mean ± SD (bar). *, **, and *** indicate *P*<0.05, 0.01, and 0.001, respectively.

To determine whether an association existed between the severity of lung injury and the tissue distribution of A/H7N9 virus, the distribution of the virus was followed at different time points after viral inoculation. The results demonstrated that many bronchial epithelial cells, but few pneumocytes or terminal bronchiole cells, were infected on day 1 postviral infection in either mouse (Fig. 4A–B). On day 5 postviral infection, antigen-positive cells appeared in the bronchioles, bronchioles terminalis, and lung tissues of both mice, but there were more of these cells in C57BL/6 mice than in BALB/c mice (Fig. 4C–D). On day 7 postviral infection, the tissue distribution of the virus in C57BL/6 mice was similar to that on day 5, whereas in BALB/c mice, only necrotic cells were observed in the bronchial epithelium and antigen distribution was limited to the interstitial tissue of the lungs (Fig. 4E–F). No virus antigen was observed in either mouse strain at 14 days postviral infection (Fig. 4G–H). The viral titers in the lung tissues were then measured. As shown in Figure 4I, viral titers in both groups increased after infection, reaching a peak on day 3 in BALB/c mice and on day 5 in C57BL/6 mice. Although viral titers dropped dramatically thereafter in both BALB/c and C57BL/6 mice, the drop in the lungs of BALB/c mice was greater than that in the lungs of C57BL/6 mice (Fig. 4A–H). Thus, H7N9 virus could replicate equally well in the lungs of both mouse strains, but BALB/c mice were more proficient in viral clearance than C57BL/6 mice, as evidenced by the earlier clearance of the virus.

### Levels of proinflammatory cytokines and cytokines in C57BL/6 mice and BALB/c mice infected with H7N9

**Figure 5:**
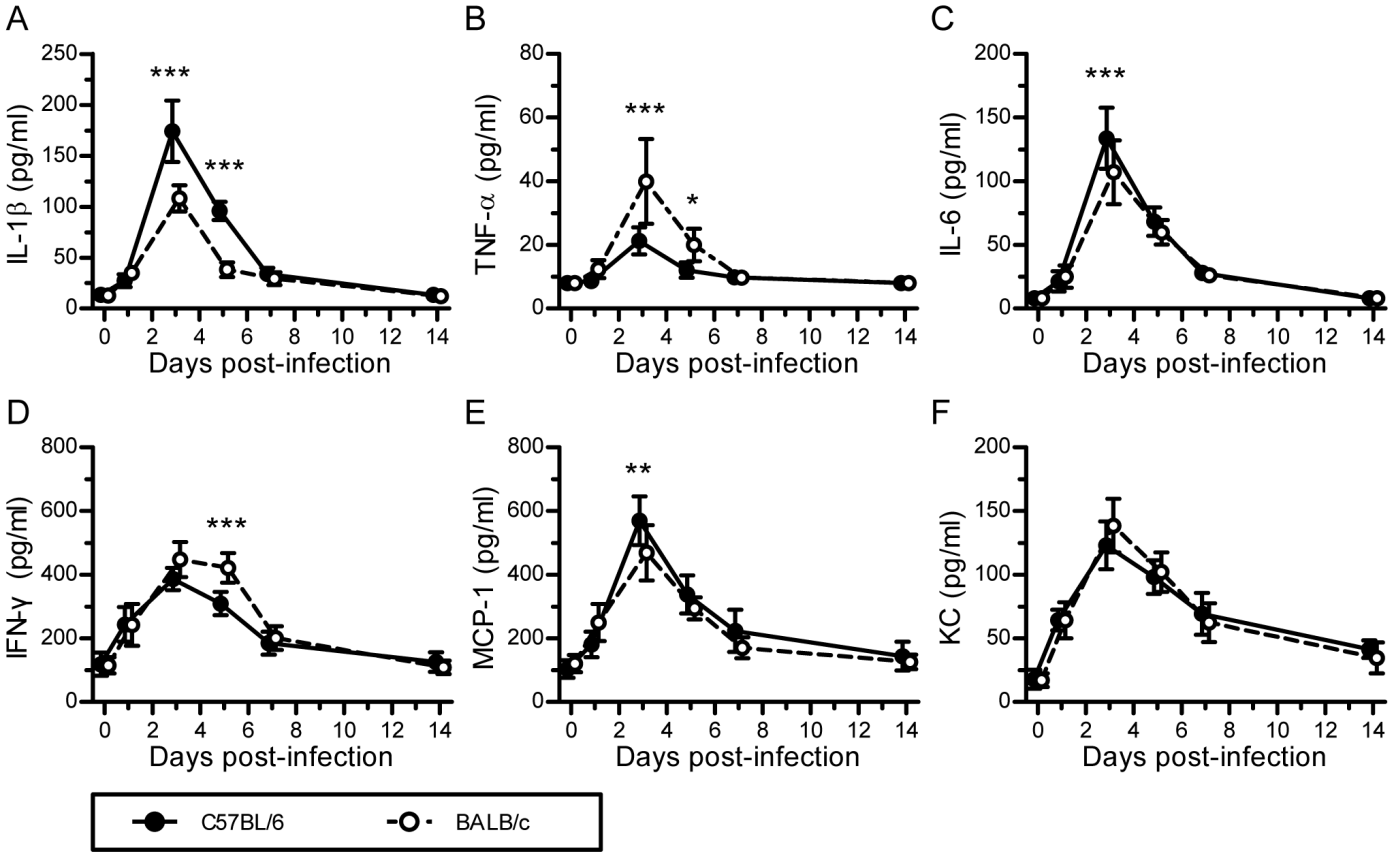
Innate immune responses induced by H7N9 virus infection in C57BL/6 and BALB/c mice. The experimental setup was the same as that described in Figure 2. (A–F) Levels of IL-1 β, TNF-*α*, IL-6, IFN-γ, MCP-1, and KC in mouse sera on days 0, 1, 3, 5, 7, and 14 postviral infection, which were measured by ELISA (*n* = 6). The data are expressed as the mean ± SD (bar). *, **, and *** indicate *P*<0.05, 0.01, and 0.001, respectively.

The dysregulation of proinflammatory cytokines and chemokines, which is sometimes referred to as a cytokine storm, is associated with lung injury and pneumonia in H7N9-infected patients [5]–[7]. To investigate whether this was also the case for the two mouse strains used in this study, we measured the levels of several representative proinflammatory cytokines and chemokines in the sera of C57BL/6 and BALB/c mice following A/H7N9 virus challenge. The results showed that the serum levels of cytokines and chemokines were significantly different on days 3 and 5 postviral infection in C57BL/6 and BALB/c mice, although their levels increased similarly in both mouse strains, reaching a peak on day 3 after H7N9 virus infection. In particular, on day 3, the levels of cytokines IL-1β and IL-6 were significantly higher in C57BL/6 mice than in BALB/c mice (Fig. 5A and C), whereas the levels of TNF-α and IFN-γ were significantly higher in BALB/c mice than in C57BL/6 mice on days 3 and 5, respectively (Fig. 5B and D). On day 3, the level of chemokine MCP-1 was also significantly higher in C57BL/6 mice than in BALB/c mice (Fig. 5E). However, there was no significant difference in the serum concentration of KC between C57BL/6 and BALB/c mice (Fig. 5F). Thus, these results suggest that lung injury was more severe and occurred more slowly in H7N9-infected C57BL/6 mice than in H7N9-infected BALB/c mice, which was probably a consequence of the more dysregulated immune response in H7N9-infected C57BL/6 mice.

### Levels of neutrophil infiltration in the lungs of H7N9-infected C57BL/6 mice and H7N9-infected BALB/c mice during the early stage of infection

**Figure 6:**
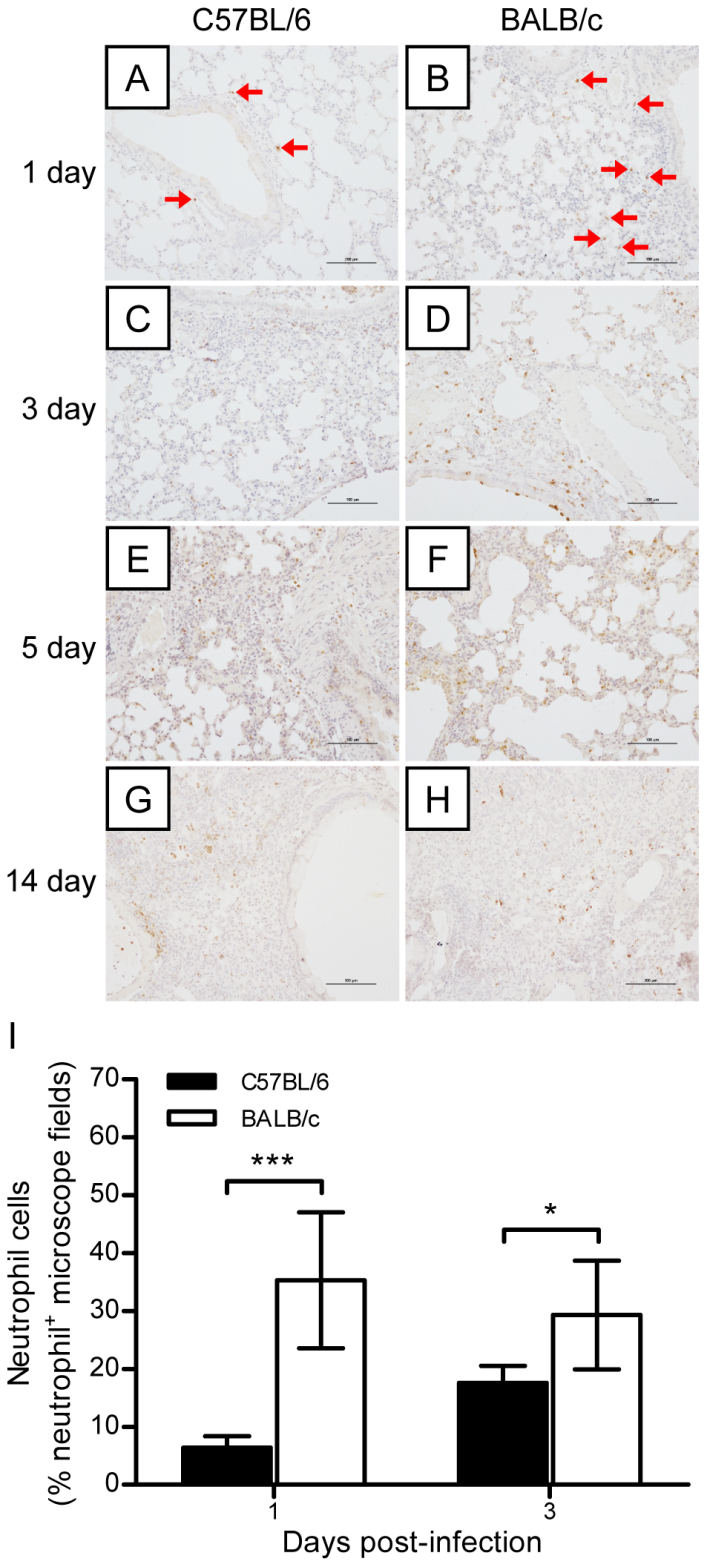
Dynamic changes in neutrophil infiltration in the lung tissues of mice after H7N9 virus challenge. The experimental setup was the same as that described in Figure 2. (A–H) Immunohistochemical staining of neutrophils in the lungs of BALB/c and C57BL/6 mice on days 1, 3, 5, and 7 postviral infection. The red arrows indicate infiltrating neutrophils. (I) Semiquantitative assessments of neutrophil infiltration in the lungs on days 1 and 3 postviral infection (*n* = 6). The data are expressed as the mean ± SD (bar). * and *** indicate *P*<0.05 and 0.001, respectively.

Neutrophil infiltration was observed as early as day 1 and it reached a peak on day 5 after A/H7N9 virus infection. Neutrophils were present mainly in the parenchyma around bronchi on days 1 and 3, whereas they were found mainly in the necrotic areas of lungs on day 5 postviral challenge. Neutrophil infiltration disappeared at 14 days postviral infection. During the early stage of viral infection, however, more neutrophils were observed in the parenchyma around the bronchi and lung tissues of BALB/c mice than in the parenchyma of C57BL/6 mice, whereas there was no significant difference in this respect between the two mouse strains on day 14 (Fig. 6). These results suggest that appropriate neutrophil infiltration, especially during the early stage of H7N9 virus infection, may be critical for suppressing virus replication and enhancing viral clearance.

### Lung lesions and virus replication in young and middle-aged mice

**Figure 7:**
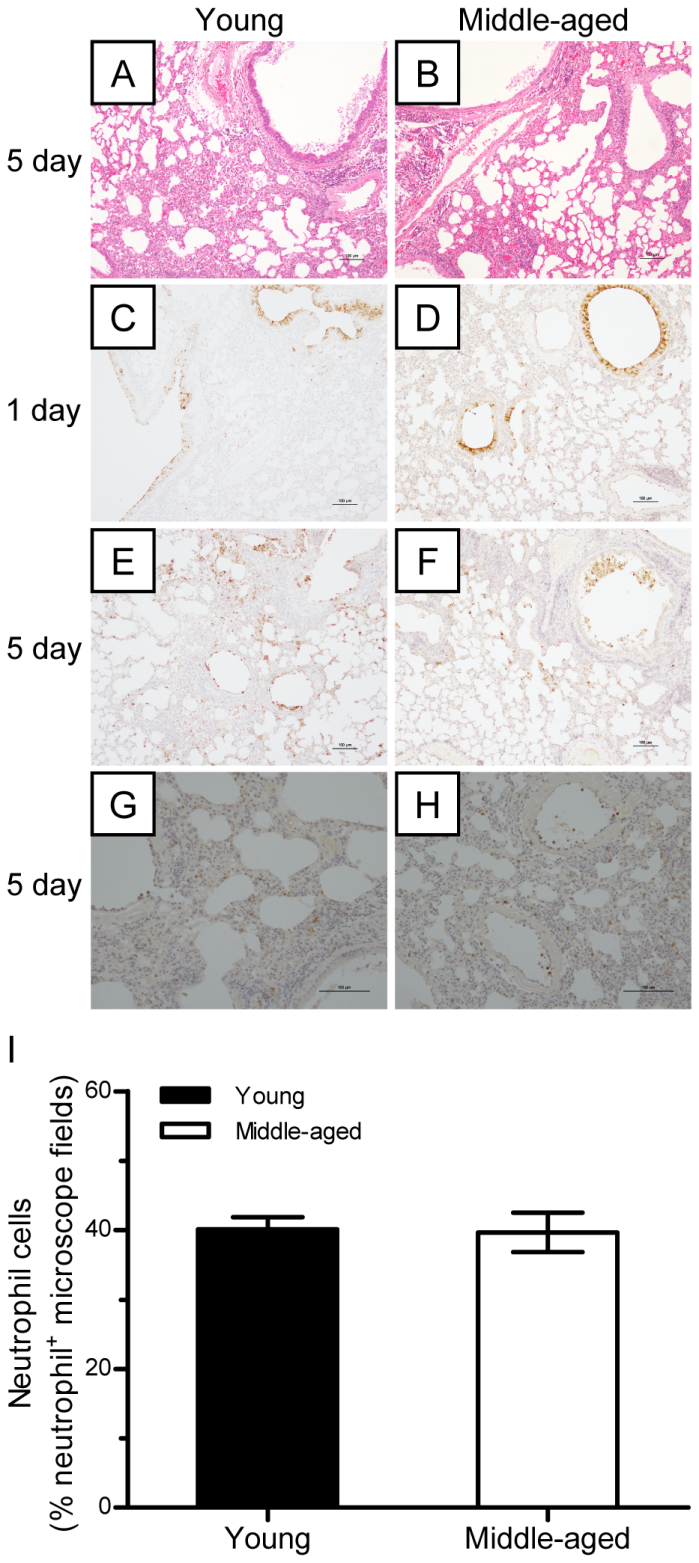
Lung lesion and virus replication in young and middle-aged mice. (A–B) Young and middle-aged BALB/c mice were challenged intranasally with 10^6^ TCID_50_ virus and euthanized at days 1 and 5 postviral infection. (A–B) Histological analysis of the lung tissues of mice on days 1 and 5 postviral infection. (C–F) Virus antigen distribution in the lungs on days 1 and 5 postviral infection (n = 5). (G–I) Neutrophil infiltration in the parenchyma of the lungs on days 1 and 5 postviral infection (*n* = 5).

To clarify whether the clinical severity of lung injury in elderly patients is related to virus replication, we examined the lung lesions in healthy naïve young and middle-aged BALB/c mice infected with H7N9 virus after H&E staining. The lung tissue distribution of the H7N9 virus was also followed by the HA antigen detection assay. The results showed that lung lesions were similar in middle-aged and young mice on day 5 postviral infection (Fig. 7A–B). The HA viral antigen was found mainly in bronchi on day 1 postviral infection, with patches appearing in the lung parenchyma on day 5 (Fig. 7C–F). There were no apparent differences in the distribution of the HA antigen or in the level of neutrophil infiltration between young and middle-aged mice (Fig. 7G–I). These results suggest that more serious lung injury in middle-aged or older H7N9 cases is not mainly caused by differences in virus replication in the lungs, but instead, it is probably a consequence of a dysregulated immune response induced by underlying comorbidities.

## Discussion

The novel avian influenza H7N9 virus has caused outbreaks of influenza in China, which have resulted in 139 infected cases and a high case fatality rate (32%) [1], [2]. Most of the patients died of severe pneumonia and ARDS with respiratory failure [6], [7]. A cytokine storm, or the upregulation of proinflammatory cytokines and chemokines, such as IL-6, IL-8, IFN-α, IP-10, MIG, MIP-1β, and MCP-1, is reported to have contributed to the lung injury and severe pneumonia in these H7N9-infected patients [5], [7]. H7N9-infected BALB/c mice produced higher levels of proinflammatory cytokines and chemokines, including IP-10, TNF-α, MCP-1, MCP-3, IFN-α, MIP-1β, KC, and regulated upon activation normal T cell expressed and secreted (RANTES), at an early stage of infection (day 3 post-infection) than BALB/c mice infected with the H9N2 virus, but lower levels than those produced when BALB/c mice were infected with H5N1 [8]. H7N9 virus replicates effectively in the nasal cavity and lung tissues of BALB/c mice, but its infection causes mild focal inflammation, with minimal involvement of the bronchial or bronchiolar epithelium [8]. These results suggest that H7N9 virus can replicate and cause disease in mice, but that it is less pathogenic than H5N1 virus.

Previous reports show that BALB/c and C57BL/6 mice differ in their susceptibility and immune responses to infection by HPAI H5N1 and pandemic H1N1 (pH1N1) viruses [10]. In this study, we performed a series of experiments to compare the pathogenicity of H7N9 infection in BALB/c and C57BL/6 mice. Despite causing severe disease and even fatal outcome, different isolates of avian influenza A H7N9 virus may have a little bit of difference on pathogenicity. Zhou et al. [7] reported a more productive replication of Anhui/1/H7N9 in lung tissues than that of Shanghai/1/H7N9 was observed. In our experiments, C57BL/6 and BALB/c mice were infected with Anhui/1/H7N9 virus, and C57BL/6 exhibited a more severe body weight loss than BALB/c mice (35% *vs* 25%, Fig. 1A). The gross lesion areas in the lungs of H7N9-infected C57BL/6 mice were significantly larger than those in the lungs of H7N9-infected BALB/c mice (45% *vs* 28%, Fig. 1B). The lung damage in C57BL/6 mice at days 5 and 7 postviral infection was more severe than that in BALB/c mice (Fig. 2), and the lung injury recovery time was longer in H7N9-infected C57BL/6 mice than in H7N9-infected BALB/c mice (Fig. 3). The numbers of viral antigen-positive cells in the bronchioles and lungs of both strains of H7N9-infected mice, as well as the viral titers in their lungs, were similar during the early stage of viral infection (1–3 days postviral infection). However, the decline in the number of virus-infected cells and viral titers was more rapid in the lungs of H7N9-infected BALB/c mice than in the lungs of H7N9-infected C57BL/6 mice (Fig. 4), suggesting that the immune system of BALB/c mice is more proficient in clearing H7N9 virus.

It is widely believed that proinflammatory cytokine and chemokine dysregulation, referred to as a cytokine storm, contributes to lung injury and severe pneumonia during the progression of a viral infection. In the present study, we compared the levels of proinflammatory cytokines in C57BL/6 and BALB/c mice infected with A/H7N9 at different time points after viral infection. We found that the levels of proinflammatory cytokines, including IL-6, MCP-1, IL-1β, TNF-α, and IFN-γ, increased immediately after infection and peaked on day 3 postviral infection, before returning to their baseline levels about 7–10 days after infection in both mouse strains. However, A/H7N9-infected C57BL/6 mice exhibited higher levels of IL-6, MCP-1, and IL-1β, but lower levels of TNF-α and IFN-γ, than A/H7N9-infected BALB/c mice at day 3 postviral infection (Fig. 5). These findings suggest that dysregulated cytokine expression may mediate lung injury in H7N9-infected hosts. Zhou et al. [7] reported that, in the absence of any other differences between the two groups, the levels of IL-6 and MCP-1 are significantly higher in H7N9- and H5N1-infected patients than in healthy subjects. Thus, it was suggested that hypercytokinemia in H7N9-infected patients may increase clinical severity, as observed previously in patients infected by H5N1 virus [3], [4].

MCP-1 plays an important role in the inflammatory and fibrotic responses to influenza A virus infection under neonatal hyperoxia [15], and van Helden et al. [16] demonstrated that the migration of NK cells to the site of influenza virus infection is MCP-1-dependent. IL-6 is an important mediator of the acute phase response and fever because it crosses the blood-brain barrier [17] and promotes PGE2 synthesis in the hypothalamus, resulting in a change in the body temperature set point. The overexpression of IL-6 during the early stage of H1N1 infection has been reported to contribute to airway inflammation and bronchial hyper-reactivity in infected children [18]. To the best of our knowledge, the present study is the first to report that the serum IL-1β level is increased in H7N9-infected mice during the early stage of H7N9 infection and that it is higher in H7N9-infected C57BL/6 mice than in H7N9-infected BALB/c mice (Fig. 5A). However, the role of IL-1β in the pathogenesis of H7N9 infection remains controversial. Schmitz et al. [19] showed that IL-1α/β mediated acute pulmonary inflammatory pathology and increased the survival rate of influenza virus-infected mice. They suggested that IL-1β may enhance IgM antibody responses and the recruitment of CD4+ T cells to viral infection sites. However, although the levels of cytokines were increased after H7N9 virus infection, the post-infection serum levels were much lower than those observed after HPAI H5N1 virus infection, especially on day 3 [8], which may explain why lung damage and illness are less severe in clinical H7N9 virus-infected patients. In the present study, the cytokine expression levels of BALB/c mice were different from those of C57BL/6 mice, which might explain the difference in lung lesions between the two mouse strains.

TNF-α, which is generally considered to be a proinflammatory and proimmune cytokine, plays an important role in the early stages of host defense against influenza infection [20], [21]. In addition, TNF-α has an immune regulatory function that controls Th1 cell activation and immunopathology following pulmonary mycobacterial infection [22], and it is also a negative regulator of CD4 and CD8 T-cell responses to lymphocytic choriomeningitis virus infection [23], [24]. Recently, Damjanovic et al. [25] reported that TNF-α was critical for controlling the extent of lung immunopathology, particularly when influenza viral clearance was near completion and lung homeostasis needed to be restored during the later phases of infection. Furthermore, intrinsic TNF/TNFR2 interactions fine tune the immune response to influenza virus infection in the lung via modest enhancements of effector functions that may function to limit the damage that could be caused by a prolonged and inefficient effector response [26]. IFN-γ, which is produced by NK cells and/or Th1 cells, enhances overall development of cell-mediated immunity, macrophage activation, antigen presentation, and chemokine gene expression. IFN-γ is a key mediator of the effect of inactivated parapoxvirus ovis against HBV and HSV infections, and the neutralization of IFN-γ by anti- IFN-γ antibodies abolish its *in vivo* antiviral effects [27]. More recently, IFN-γ was shown to play a pivotal role in controlling virus infection and in resolving acute inflammation [28], [29]. In the present study, the lower level of lung damage as well as the more effective and earlier recovery of BALB/c mice may be due to the higher levels of TNF-α and IFN-γ in the serum of BALB/c mice on days 3 and 5.

Neutrophils, which migrate readily into lung tissues in response to inflammatory stimuli, occur at higher levels in pulmonary capillaries than in systemic blood. Thus, neutrophils are considered to be the host immune system's first line of defense against infection. Tumpey et al. [30] demonstrated that neutrophils and alveolar macrophages control the replication and spread of a lethal recombinant influenza virus bearing 1918 H1N1 virus HA and NA, because the depletion of neutrophils and alveolar macrophages before viral challenge resulted in uncontrolled virus replication and increased mortality in mice. Tate et al. [31] also showed that neutrophils could limit virus replication and ameliorate lung injury. Furthermore, Fujisawa [32] reported that neutrophils protect against and aid recovery from influenza virus infection in the lungs of mice. In the present study, we showed that neutrophil infiltration increased during the early stage of A/H7N9 virus infection, but decreased during the later stage, in both C57BL/6 and BALB/c mice. The lower level of neutrophil infiltration during the early stage of infection in the lungs of C57BL/6 mice may be one of the reasons why H7N9-infected C57BL/6 mice exhibited more severe pulmonary damage and slower lung injury recovery than H7N9-infected BALB/c mice.

Epidemiological studies of human A/H7N9 virus infections show that the most severe cases occur among middle-aged and elderly patients and the age distribution differs from that of HPAI H5N1-infected cases [33], [34]. However, it is still unclear whether severity of lung injury in middle-aged or older cases is immediately related to more viral replication, or to the activity of host factors after viral infection. In the present study, we found that there was no significant difference in HA antigen distribution between young and middle-aged mice during the first few days after infection, indicating that young and middle-aged mice have similar susceptibility to H7N9 virus infection. Thus, more serious lung injury in older H7N9 cases is probably attributable to differences in the host immune response. The immune responses to pathogen invasion differ with age and aged patients are considered to be at an increased risk for influenza complications because of age-related deceases in immunity or underlying medical conditions [35]–[37]. In the present study, the lung lesions were similar between healthy influenza-naïve BALB/c young and middle-aged mice on day 5 postviral infection. Thus, the severe nature of the disease in aged patients is probably due to an existing dysregulated immune response induced by underlying medical conditions.
